# Candidate Gene Markers Associated with Fecal Shedding of the Feline Enteric Coronavirus (FECV)

**DOI:** 10.3390/pathogens9110958

**Published:** 2020-11-17

**Authors:** Jana Bubenikova, Jana Vrabelova, Karla Stejskalova, Jan Futas, Martin Plasil, Petra Cerna, Jan Oppelt, Dana Lobova, Dobromila Molinkova, Petr Horin

**Affiliations:** 1 Department of Animal Genetics, Faculty of Veterinary Medicine, University of Veterinary and Pharmaceutical Sciences Brno, 61242 Brno, Czech Republic; bubenikovaj@vfu.cz (J.B.); stejskalovak@vfu.cz (K.S.); JFutas@VFU.cz (J.F.); plasilma@vfu.cz (M.P.); 2 Department of Infectious Diseases and Microbiology, Faculty of Veterinary Medicine, University of Veterinary and Pharmaceutical Sciences Brno, 61242 Brno, Czech Republic; VrabelovaJana@seznam.cz (J.V.); petra.cerna@colostate.edu (P.C.); lobovad@vfu.cz (D.L.); molinkovad@vfu.cz (D.M.); 3 CEITEC VFU, RG Animal Immunogenomics, University of Veterinary and Pharmaceutical Sciences Brno, 61242 Brno, Czech Republic; jan.oppelt@gmail.com; 4 Department of Clinical Sciences, Colorado State University, Fort Collins, CO 80523-1678, USA; 5 Department of Pathology and Laboratory Medicine, Division of Neuropathology, Perelman School of Medicine, University of Pennsylvania, Philadelphia, PA 19104-6100, USA

**Keywords:** feline enteric coronavirus, fecal shedding patterns, polymerase-chain reaction, genetic susceptibility, immunity-related candidate genes, association study

## Abstract

The Feline coronavirus (FCoV) can cause a fatal disease, the Feline Infectious Peritonitis. Persistent shedders represent the most important source of infection. The role of the host in FCoV fecal shedding is unknown. The objective of this study was to develop gene markers and to test their associations with FCoV shedding patterns. Fecal samples were taken from 57 cats of 12 breeds on the day 0 and after 2, 4 and 12 months. Variation from persistent and/or high-intensity shedding to no shedding was observed. Thirteen immunity-related genes were selected as functional and positional/functional candidates. Positional candidates were selected in a candidate region detected by a GWAS analysis. Tens to hundreds of single nucleotide polymorphisms (SNPs) per gene were identified using next generation sequencing. Associations with different phenotypes were assessed by chi-square and Fisher’s exact tests. SNPs of one functional and one positional candidate (*NCR1* and *SLX4IP*, respectively) and haplotypes of four genes (*SNX5*, *NCR2*, *SLX4IP*, *NCR1*) were associated with FCoV shedding at *p*_corected_ < 0.01. Highly significant associations were observed for extreme phenotypes (persistent/high-intensity shedders and non-shedders) suggesting that there are two major phenotypes associated with different genotypes, highly susceptible cats permanently shedding high amounts of viral particles and resistant non-shedders.

## 1. Introduction

The Feline coronavirus (FCoV) is ubiquitous in feline populations worldwide, and despite being first described in 1963 [1], it remains one of the most poorly understood feline viruses. The virus belongs to the order *Nidovirales*, family *Coronaviridae*, subfamily *Orthocoronavirinae*, and genus *Alphacoronavirus* 1. Two primary forms (biotypes) of FCoV are recognized: the avirulent enteric form (Feline Enteric Coronavirus (FECV)) and the virulent form (Feline Infectious Peritonitis Virus (FIPV)) causing a fatal clinical disease, feline infectious peritonitis (FIP). Infection with FCoV is a major problem in multiple-cat households and, to a lesser extent, in free-roaming cats. The virus is endemic especially in multi-cat environments, where many cats are kept together in a small space, such as catteries and shelters. Infection with FCoV is common in domestic cats, with up to 90% of cats within multi-cat households being seropositive [2]. The majority of FCoV infections are asymptomatic or are associated with mild intestinal disease; however, approximately 1–5% of infected cats develop FIP [3,4], which is characterized by the development of a variable combination of pyogranulomatous polyserositis, vasculitis and granulomatous lesions in organs, and an extremely high mortality rate [5,6].

The FECV infection is spread mostly by the orofecal route [7,8], while other modes of transmission are rather rare [4,9]. Fecal shedding of the virus usually begins within one week of exposure [10]. The primary stage of infection after exposure to FCoV can last from 7 to 18 months. During this period, the highest levels of viral shedding are observed [8]. In naturally infected cats, the initial shedding levels decrease over two years [11]. Cats may shed FECV with low, moderate or high frequency. Shedding usually persists for 4 to 6 months, followed by intermittent shedding and eventual clearance [12]. In some cats, FECV shedding may persist for more than 18 months [8].

The patterns of fecal shedding of FECV may differ between single-cat and multi-cat households [13]. A large proportion of cats in multiple-cat environments undergo cycles of infection and shedding, recovery, and reinfection, shedding the virus the entire time [7]. More than 80% of cats living in catteries shed FECV while being clinically asymptomatic [14]. In multi-cat households, persistent (10–15%), intermittent (70–80%) or self-limiting FECV shedding patterns were observed; about 5% of cats were identified as non-shedders [15]. Based mostly on unpublished data, Addie et al. [16] recently discussed the possibility that intermittent FECV shedding may in fact be false negativity due to technical reasons. It was suggested that identification of long-term carriers requires multiple positive results for at least eight consecutive months, while a cat could be considered negative with multiple negative results over a period of 5 months [15]. Persistent shedders can be infected with the same strain of the virus the whole time, but reinfection after recovery with the same or a different virus strain has also been reported [11,17].

Asymptomatic persistent shedders represent the most important source of infection. They were identified as a source of infection for susceptible kittens [4]. Host factors such as age, environmental stress, concurrent disease and especially various types of immune responses play an important role in the development of FIP [18]. Genetics represents one of the factors contributing to the observed variation in the course of coronavirus infections, including the shedding status in humans [19]. In cats, GWAS candidate regions and several individual candidate genes were observed to be associated with FIP [20,21]. Much less is known about the role of the host in FECV fecal shedding. Interbreed differences in the presence of anti-FCoV antibodies in healthy cats have been reported repeatedly [13,22,23]. However, as some of these differences were observed between purebred and crossbred cats from different environments, it is not clear to what extent they might be due to non-genetic factors, such as the intensity of close contacts. Addie et al. [17] genotyped the feline Major Histocompatibility Complex (*MHC*) *FLA* class II *DRB* genes in 25 cats for which fecal shedding status was determined. They found no statistical association with the MHC genes analyzed.

Studies of the variation in the shedding status of FECV can contribute to the understanding of its role in the spread of coronavirus infections among domestic cats. Identification of genes involved in phenotypic variation of complex traits including infections may contribute to the identification of mechanisms underlying the phenotypes observed [24]. Finally, strong phenotype/genotype associations may be considered for identification of cats susceptible to massive virus shedding.

In a broader context, the current COVID-19 pandemic raises questions about similarities between coronaviruses infecting different mammalian species including pets. As non-symptomatic SARS-CoV-2 shedders represent a serious epidemiological threat, mechanisms of coronavirus shedding merit attention. Cats have been reported to be susceptible to subclinical infection with SARS-CoV-2, being able to shed the virus through oral and nasal secretions without any clinical signs and to transmit the virus to other cats by direct contact [25,26]. Considering our so far rather fragmented knowledge about SARS-CoV-2 infection, it is not clear to what extent human and cat coronaviruses are similar or differ. Information retrieved from animal models including cats can be helpful for the current COVID-19 research [27].

Therefore, the objectives of this study were (i) to determine patterns of one-year fecal shedding of FECV in a cohort of cats in multi-cat households with different pure breeds, (ii) to define phenotypes suitable for analyzing the genetic variation underlying the variation in the shedding status, (iii) to identify and characterize candidate genes and their polymorphic markers potentially associated with the shedder status and (iv) to test associations between candidate polymorphic markers and FECV shedding in the cohort analyzed.

## 2. Results

### 2.1. Phenotyping: Determination of FECV Shedding Patterns

#### 2.1.1. Shedding Patterns

Based on qPCR detection of FCoV in four samplings, four patterns/phenotypes were identified.

1. Non-shedders (NS): cats with all four samples negative: *n* = 7. These cats were not detected as shedders in any qPCR over the period of 12 months.
2. Intermittent shedders (IS): cats with at least one positive and one negative sampling followed by another positive sampling: *n* = 15. For these shedders, it was clear that they stopped and re-started shedding at least once.
3. Shedders with unclear status (US): cats for which the shedding patterns could not be determined based on four samplings: *n* = 21. In this category of shedders, the first or the fourth samples was negative, combined with uninterrupted shedding in one, two or three consecutive samples. This category thus may include potential intermittent shedders, persistent shedders and self-limiting cats, which stopped shedding. Based on the four samples analyzed, it was not possible to predict which pattern would be observed in the future.
4. Persistent shedders (PS): cats with all four samples positive (*n* = 14). These cats were detected as shedders of the virus over the whole experiment.

#### 2.1.2. Intensity of Shedding

Based on the total numbers of virus particles detected in each cat over the period of 12 months, five non-overlapping semi-quantitative categories were identified and considered as phenotypes for association analyses. Real values observed within each phenotype document the differences between them.

1. Non-shedders (NS): no virus detected by qPCR (*n* = 7).
2. Very low-intensity shedders (VLS): 1–1000 virus particles (*n* = 4).
  Real values within this category: 370–620.
3. Low-intensity shedders (LS): 1000–4999 virus particles (*n* = 11).
  Real values within this category: 1180–4600.
4. Medium-intensity shedders (MS): 5000–99,999 virus particles (*n* = 24).
  Real values within this category: 10,000–87,440.
5. High-intensity shedders (HS): more than 100,000 virus particles (*n* = 11).
  Real values within this category: 105,480–2,177,500.

No difference in the intensity of shedding was observed between males and females as assessed by the Mann–Whitney test (*p* > 0.05).

### 2.2. Candidate Genes and Their Molecular Markers

Numbers and other characteristics of SNPs found by next generation sequencing in all candidate genes are in Table 1. More detailed information about SNPs is available in the Mendeley repository https://data.mendeley.com/datasets/cxx68cw77c/draft?a=ac846404-9385-424e-890f-62484981eb64.

A list of haplotypes undoubtedly identified based on NGS data is in Supplementary Table S3.

MAFs and heterozygosities calculated for the study cohort and their comparisons with population characteristics of stray cats are in Supplementary Materials Table S1. The data showed that the variation of the multi-breed cohort was to some extent different from the stray cat group in terms of MAFs as well as for heterozygosity estimates. However, for 19 out of the 21 (90.5 %) most significant SNPs associated with FECV shedding, neither MAFs nor heterozygosities were different between these two groups (Supplementary Materials Table S1).

### 2.3. Association Analyses

Out of the 13 candidate genes analyzed for associations by Chi-square and Fisher’s exact tests with Bonferroni corrections, nine genes (*NCR1*, *NCR2*, *NCR3*, *IFNB1*, *MHC-DRA*, *MHC-DRB*, *SNX5*, *SLX4IP*, *PCSK2*) showed associations of single SNPs or their haplotypes with the persistency or intensity of FECV shedding at *p*_uncorrected_ < 0.01 (Table 2, Supplementary Table S4).

Markers of three of these genes (*DRB, PCSK2* and *SLX4IP*) were associated with both kinds of phenotypes (Table 2). Markers selected for further analyses and the corresponding associations which remained significant after Bonferroni corrections are in Table 3 and Supplementary Table S5.

For the most significant associations analyzed in detail, markers of all seven genes associated with shedding intensity were associated with high intensity of shedding (HS) when compared to other phenotypes; a marker of the *MHC-DRB* gene was associated with no shedding (NS) (Table 3). All associations with shedding patterns concerned the non-shedder (NS) or persistent shedder (PS) phenotypes in comparison with other phenotypes or their combinations (Table 3).

Out of the nine most significant associations, eight concerned three SNPs within the *SLX4IP* gene. Among them, six involved the PS phenotype, and two associations involved the HS phenotype, both of them in comparison with various other phenotypes or combinations thereof. The one highly significant association observed for the *NCR1* gene also involved HS in comparison with a combination of other phenotypes (Table 4). A summary of SNP markers significantly associated at *p*_corrected_ < 0.01 with particular phenotypes is in Table 4. The most significant associations were observed for genes *NCR1* and *SLX4IP*, at *p* < 0.005 and 0.001, respectively. Six out of nine loci associated with FECV shedding were in Hardy–Weinberg equilibrium (Supplementary Table S5).

A summary of haplotypes significantly associated at p_corrected_ < 0.01 with particular phenotypes is in Table 5. Out of 14 haplotypes of four genes associated with FECV shedding at p_corrected_ < 0.01, nine involved persistent shedders (PS), five of them in comparisons with non-shedders. The remaining five haplotypes were associated with the high intensity of shedding (HS) phenotype compared to various combinations of other phenotypes (Table 5).

Composed SNP genotypes were associated with both kinds of phenotypes and showed synergies especially between the *NCR1* and *NCR2* genes, as well as strong associations of physically linked genes (Table 6 and Table 7).

Out of the four composed intragenic genotypes with the most significant phenotype associations, two associations involved the HS phenotype, one concerned the PS phenotype, and one concerned no shedding (NS); in each case the above-mentioned phenotypes were compared to combinations of other phenotypes. In two of these cases, NS was part of a group of merged no or low shedder phenotypes (Table 6) compared to combinations of other phenotypes.

Two-gene genotypic combinations of functionally related *NCR1*, *NCR2* and *NCR3* markers and of physically linked GWAS positional markers were strongly associated with different shedder phenotypes at *p* values ranging from 0.008 to 0.00004 (Table 7, Supplementary Table S6). Out of 6 composed intergenic genotypes presented in Table 7, three involving positional markers were associated with persistent shedding, while three involving the *NCR 1* and *2* genes were associated with the HS phenotype.

The results showed that polymorphic markers located within a candidate genomic region of chromosome A3 associated with FIP in a previous GWAS [21] were associated with fecal shedding of FECV. As for functional candidates, genes underlying innate immunity mechanisms, in particular the function of NK cells, were significantly associated with the phenotypes studied.

A complete list of alleles/genotypes associated with susceptibility to persistent/high-intensity shedding is in Supplementary Table S7.

## 3. Discussion

The results of association analyses primarily depend on the phenotypes defined. Based on the parameters of specificity and sensitivity, we are confident that our qPCR assay detected cats shedding FECV with sufficient accuracy. All cats studied were from multi-cat households with at least two cats in close contact, sharing the same toilet. Within all groups, at least one cat in each household was shedding FECV. Therefore, we believe that all cats were exposed to infection. The observed dynamics of shedding patterns are in agreement with this assumption, although the infection pressure was necessarily not the same between different catteries. We have observed similar shedding patterns to those identified by Addie et al. [15] in a similar type of study. In terms of persistency of shedding, different types of shedders along with non-shedders were observed. For cats with three or fewer consecutive samplings positive for FECV (37%), it was not possible to determine the shedder status unequivocally, and therefore, this group was analyzed separately, as a special phenotype. Despite possible methodological variables during sample preparation and processing, the estimated numbers of virus particles in individual samples provided by the qPCR allowed us to distinguish non-overlapping groups of cats, which we analyzed as distinct semi-quantitative phenotypes (Supplementary Table S8).

Significant associations of candidate genes with both persistency and intensity of shedding were observed. An overall comparison across the two categories of phenotypes showed that a vast majority of the most significant associations observed for individual SNPs and haplotypes as well as for intragenic and intergenic composed genotypes involved single extreme phenotypes, i.e., persistent shedders (PS) and/or high-intensity shedders (HS), on one hand, and non-shedders (NS), on the other hand, in comparison with various other phenotypes or their combinations (Table 3, Table 4, Table 5, Table 6 and Table 7). These single phenotypes often shared alleles/genotypes (individual data are available in the Mendeley repository). Based on these results, it seems that the candidate genes studied here influence especially host susceptibility to virus infection expressed by the phenotypes PS and HS, and host resistance manifested as the NS phenotype. Only rarely were significant associations with the candidate genes observed for the remaining “intermediate” phenotypes defined as single phenotypes (as opposed to when merged with other phenotypes). We thus may speculate that the observed variation in shedding patterns could primarily result from a quantitative variation in the intensity of shedding and that there are two major phenotypes associated with different genotypes, i.e., resistant non-shedders and highly susceptible cats permanently shedding high amounts of viral particles. Interestingly, for some SNPs within the *SLX4IP* gene, heterozygotes shed fewer virus particles than homozygotes (Supplementary Table S7), indicating a possible advantage of heterozygosity of the corresponding GWAS candidate genomic region. Cats of average resistance/susceptibility to FECV infection would then represent a range of variation between these two extreme phenotypes, influenced not only by the underlying genetic variation but also by a variety of non-genetic factors, as was observed for infection with FIPV and for clinical FIP [18]. This assumption is in agreement with the idea of non-existence of intermittent shedders [16].

Biological interpretations of statistical association studies have several limitations. The cohort analyzed here is a heterogeneous multi-breed group with two prevailing breeds, Maine Coon and British Shorthair, and composed of cats of different ages, some of them related. Pure breeds are under strong pressure of artificial selection and are generally more homozygous than for example crossbred stray cats (Supplementary Materials Table S1). As such, the group studied is structured, which may have led to false positive results. However, it proved to be impossible to establish a sufficiently large group matching exactly the purposes of this experiment. The composition of the group analyzed was primarily established by breeders willing to participate in such a long-term study. We thus had only limited chances to control breed, age and other factors important for an ideal statistical design. On the other hand, a comparison with a group of stray cats, which are supposed to represent a wide range of intraspecies variation, showed that for associated markers, their minor allele frequencies (MAFs) were mostly similar in both groups, including the genes involved in the most significant associations. Nevertheless, we cannot exclude that some of the associations presented here are false positives. Although most of the associated markers were primarily in Hardy–Weinberg equilibrium (HWE), some of them were not. However, these differences in HWE concerned different SNPs within the same genes; most associated SNPs were in HWE except *NCR2* (Supplementary Tables S2 and S3). Although no significant associations were observed for this gene after corrections, in composed genotypes, it clearly contributed to associations, increasing the significance of the *p* values of its respective partners by one order of magnitude (Table 7, Supplementary Table S6).

We were confronted with a rather complex situation for MHC class II genes, which did not allow us to analyze associations of *DRA-DRB* haplotypes. There are at least two *DRA* loci and several *DRB* loci in cats. Non-distinction of different *DRB* loci, undetermined *DRB* genotypes, and low numbers of cats of several different breeds were probably reasons why Addie et al. [17] found no associations of FECV shedding with MHC. Similarly, our primers proved to amplify two different *DRA* loci, with one, two, three or four alleles observed per individual, and we did not succeed in designing *DRA* locus-specific primers. On the other hand, we are confident that our *DRB* primers were locus-specific, with one or two alleles observed per individual. In this situation, composed MHC *DRA/DRB* genotypes were not informative.

Despite all of these limitations, we are confident that it is reasonable to suggest that at least some of these SNP associations indicate biologically plausible effects of specific genes on the shedder phenotypes. All of them remained highly significant even after overconservative Bonferroni corrections, some of them at *p* values exceeding 0.01 by orders of magnitude. Some of them could be confirmed on even smaller numbers of cases by a within-breed analysis, reducing some of the biases. The most significant association (*p* < 0.0061) was found in the British Shorthair subgroup of cats for *NCR1*, where a SNP (#3093) was associated with differences between non-shedders/low shedders and medium/high shedders (Supplementary Materials Table S2).

Associations observed for composed SNP genotypes showing functional synergies, especially between the *NCR1* and *NCR2* genes, as well as strong associations of physically linked genes also support the hypothesis of their biological plausibility.

This hypothesis is compatible with known biological roles of functional candidates. MHC class I and II loci were associated with different coronavirus infections and their outcomes in poultry [28]. Associations with MHC loci and mortality in COVID-19 was recently reported [29].

The natural cytotoxicity receptors on NK cells have different functions in important biological processes, such as virus immunity, cancer immunity and human pregnancy [30]. The product of the human *NCR1* gene (NKp46) was shown to recognize viral hemaglutinins of various virus families, including influenza, Sendai virus, poxvirus and Newcastle disease virus [31]. NK cells seem to play important roles in human coronavirus infections of zoonotic origin [32], and they also seem to be important for FIPV infection of cats [33,34]. Our data suggest that NK cells might be important for controlling the process of FECV shedding as well.

**Figure 1:**
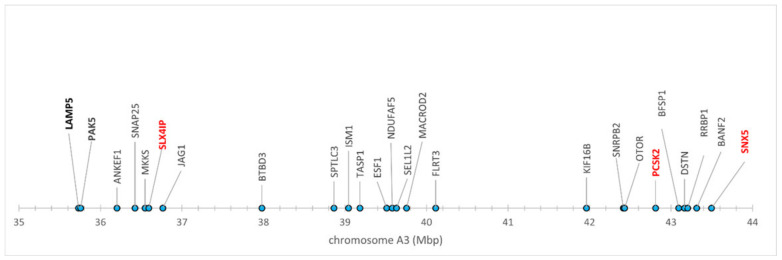
Candidate genomic region on chromosome A3. Significantly associated genes are in red. Studied genes showing no association are in bold.

On the other hand, the positional/functional candidates represented a kind of positive control of this experiment; the results obtained indicate that they are not false positives. Although they were selected based on previously reported associations of the chromosome regions, where they are located, with clinical FIP [21], the positional candidates *PCSK2, SNX5* and *SLX4IP* showed significant associations with FECV shedder phenotypes as well (Table 1, Table 2, Table 3, Table 4 and Table 5, Supplementary Tables S4 and S5). Most of these loci were in HWE (Supplementary Tables S4 and S5, information about all SNPs are in Mendeley repository). Based on their physical positions in the cat genome assembly FelisCatus v. 9.0 [35] and on the *p* values of their associations with the phenotypes studied, we could tentatively outline the candidate region on chromosome A3 (Figure 1).

The physiological functions of these primarily positional candidates do not contradict the hypothesis of their involvement in mechanisms of response to a viral infection. It was shown that the activation of the SLX4 complex during HIV infection may modify innate immune sensing and interferon production [36]. SNX5 is involved in macropinocytosis and antigen processing in macrophages [37]. The role of PCSK2 is unclear; it is a serine protease expressed in enterocytes, and coronavirus spike proteins require cleavage when entering the cell [38].

In summary, we have observed similar patterns of fecal FECV shedding in multi-cat catteries to those previously reported by other authors. So far, only genes associated with clinical disease induced by FIPV have been identified, and no information about genes underlying the variation in FECV shedding has been available. For this purpose, we have developed polymorphic functional and positional candidate gene markers, and for some of them, we have observed their non-random distribution amongst different categories of FECV shedders and non-shedders. The majority of highly significant associations were observed for extreme phenotypes. However, the phenotypes analyzed in this study are complex, as they are influenced by multiple factors, including re-infections; nonetheless, differences between high-intensity shedders, persistent shedders and non-shedders compared to various combinations of other phenotypes were distinct for several markers.

The data presented here contributed to a better characterization of shedders, the most important source of infection in multi-cat households. Known biological functions of genes associated with FECV shedding in this study support the hypothesis of the biological plausibility of the selected functional candidates. The results suggest that innate immunity and especially the role of NK cells in cat coronavirus infections, not yet studied in this context, merit further attention. Associations of positional candidate markers, i.e. SNPs located within a genomic region identified by a GWAS as a candidate for FIPV susceptibility, suggest that at least some of the immune mechanisms involved in the control of infection with FIPV also operate in the control of FECV shedding, which is a hypothesis that we are currently testing.

## 4. Materials and Methods

### 4.1. Phenotyping: Determination of FECV Shedding Patterns

#### 4.1.1. Study Design

##### Animals

A total of 57 mostly unrelated purebred cats of 12 breeds living in multi-cat households (Supplementary Table S9) were included in the study. All owners involved in the study signed an informed consent document and agreed to all related procedures. Only catteries with at least one PCR positive cat were included in the study.

The group was composed of 20 males and 37 females. The median age for this group was 23 months; the mode was 20 months, and the range was from 3 months to 14 years.

##### Sampling

Four fecal samples were taken by individual owners from each cat between April 2019–May 2020 at day 0 and then after 2, 4 and 12 months. For some individual samplings, the intervals were somewhat longer/shorter, mostly due to complications related to the COVID-19 restrictions. Feces (200–220 mg) were collected into 1 mL of the RNA stabilizer in a sterile tube; RNA later^®^ (Sigma Aldrich, St. Louis, MO, USA) was used for longer preservation of the cat feces samples. Samples were stored in a refrigerator (4 °C) until transported to the clinical laboratory.

A group of 38 stray cats consisting of cats originating from local shelters and castrated and/or cured at the UPVS Clinic for Small Animals was used for characterizing the population diversity of candidate gene markers and for comparison with the group of purebred cats. Peripheral blood samples used for DNA extraction were not collected solely for the purposes of this study but on the occasion of other veterinary interventions. Blood samples were collected at the clinic by licensed veterinarians in compliance with all legal, professional and ethical standards.

All procedures were approved by the Ethical committee of the UPVS Brno as part of the project (Ref. PP-IGA-2019-Horin).

#### 4.1.2. RNA Isolation

Total RNA was extracted from 150 mg of each fecal sample using the NucleoSpin^®^ RNA Stool (Macherey Nagel, Düren, Germany) commercial extraction kit in accordance with the manufacturer’s instructions. Total RNA was eluted in 100 μL RNase-free water and stored at −80 °C.

#### 4.1.3. Reverse Transcription

RNA (5 μL) was transcribed into cDNA form immediately after RNA isolation by using the Transcriptor First Strand cDNA Synthesis Kit (Roche, Basel, Switzerland) with Random oligonucleotides.

#### 4.1.4. Identification of FCoV: Quantitative PCR

##### Preparation of the Quantitation Standard

One part of the 3′-UTR of FCoV transcribed into cDNA was amplified with the P205/P211 primer pair GGCAACCCGATGTTTAAAACTGG/CACTAGATCCAGACGTTAGCTC [39] and cloned into the pCRTM8/GW/TOPO vector. The recombinant vector was transformed into One Shot competent *E. coli* Top10 cells (Invitrogen, Waltham, MA, USA). After its replication and purification by Purelink quick plasmid miniprep kit (Sigma Aldrich, St. Louis, MO, USA), its concentration was determined by spectrophotometry. Standard concentrations ranging from 10^7^ copies/μL DNA copies to 10^1^/μL copies of DNA were used for constructing a qPCR calibration curve. The dilution with 10^5^; DNA copies was included in each qRT-PCR reaction as a standard.

##### Quantitative PCR

Primers P204 (5′-GCTCTTCCATTGTTGGCTCGTC-3′, antisense) and P276 (5′-CCGAGGAATTACTGGTCATCGCG-3′, sense) [39] were used. Both detection and quantification of FCoV RNA were performed using Xceed qPCR SG 1step 2 × Mix Lo -ROX (Institute of Applied Biotechnologies, Prague, Czech Republic) in a total volume of 20 μL in accordance with the manufacturer’s instructions on a Light Cycler^®^ 480 II (Roche, Basel, Switzerland). Amplification conditions were initial denaturation at 95 °C/7 min, followed by 45 cycles consisting of denaturation at 95 °C/10 s and primer annealing/extension at 60 °C/30 s. Immediately after the PCR, a melting curve was created by raising the incubation temperature from 55 to 95 °C to confirm the specificity of the products obtained. Samples whose melting temperature matched the control Tm (80 °C) were considered as positive. For the positive control, 10^5^ copies of the FCoV plasmid DNA were added. PCR water was used as a negative control. The specificity and sensitivity of the reactions calculated according to standard protocols were 1.0 and 0.958, respectively, with the limit of detection set as 10^2^ copies in 1 μL; the predictive values were 1.0 for a positive result and 0.909 for negative reactions. Based on the calibration curve, estimates of the numbers of virus particles generated by the Light Cycler were recorded and used for semi-quantitative phenotyping.

Note: Taking into consideration the specificity of the primers, we used the abbreviation FCoV in situations where a distinction between the enteric and the virulent form was not a priori obvious, while FECV was used for the virus detected in feces of healthy cats and FIPV for citations of data related to clinical FIP.

#### 4.1.5. Determination of Phenotypes for Association Analyses

Two kinds of shedding pattern phenotypes were determined: persistency and intensity of shedding. Persistency was assessed based individual patterns of shedding observed throughout the entire experiment. In agreement with previous observations that a cat could be considered negative with multiple negative results over a period of 5 months and the identification of long-term carriers requires multiple positive results for at least eight consecutive months [15], we considered cats with all four samples negative in the qPCR assay over a period of 12 months as non-shedders (NS) and cats with positive qPCR for all four samples as persistent shedders (PS). Cats with at least one positive and one negative sampling followed by another positive sampling were considered as intermittent shedders (IS). For all other cats, in which it was impossible to distinguish between the intermittency of shedding and other possible patterns based on the four samplings, the shedding status was described as unclear, and they were considered as a separate phenotype (US). The intensity of shedding was evaluated based on the total numbers of virus particles estimated in all four fecal samples analyzed. Semi-quantitative patterns/phenotypes were determined as categories characterized by non-overlapping ranges of the numbers of virus particles observed within the cohort.

### 4.2. Candidate Genes and Their Molecular Markers

#### 4.2.1. Selection of Candidate Genes

Thirteen immunity-related genes were selected as functional (*IFNB1*, *IFNL1*, *IFNLR1*, *NCR1*, *NCR2*, *NCR3*, *MHC-DRA*, *MHC-DRB*) and positional/functional (*SNX5*, *SLX4IP*, *PCSK2*, *PAK5*, *LAMP5*) candidates (Supplementary Table S10). The functional candidates were selected based on hypotheses about their possible biological role in FCoV infection. Since no such analysis has so far been performed for FECV shedding, the positional candidates were selected from genes located in a candidate region detected by a previously published GWAS analysis for clinical FIP [21]. The hypothesis tested was that at least some genes associated with the control of FIPV infection also control FECV shedding.

#### 4.2.2. Bioinformatic Analysis of Candidate Genes

The most recent domestic cat genome assembly (*Felis catus*, v. 9.0, GenBank accession GCA_000181335.4) was used for identifying sequences corresponding to selected genes. Based on the length of the genes and their intron content, the sequences then served for designing primers amplifying either full length genes or their functionally important parts (Supplementary Tables S11 and S12).

#### 4.2.3. DNA Extraction

Peripheral blood was collected from all cats included in the study. Based on the informed consent signed by all owners, blood samples were always collected by a licensed veterinarian in agreement with all legal, professional and ethical standards, mostly at the occasion of vaccination and/or other veterinary interventions during the one-year study. This approach was approved by the Ethical committee of the Veterinary University Brno. DNA was extracted from 200 μL of the EDTA-anticoagulated blood samples using the NucleoSpin Blood kit (Macherey-Nagel, Düren, Germany) according to the manufacturer’s instructions.

#### 4.2.4. PCR Amplifications

PCR protocols differed according to individual genes (Supplementary Tables S11 and S13) and were created in accordance with manufacturers’ manuals. In general, the total reaction volume was 12.5 μL for all master mixes. *DRA* and *DRB* genes were amplified twice in independent PCRs.

#### 4.2.5. Next Generation Sequencing

Sequencing was done on the Illumina MiSeq sequencer using a standard flow cell and v. 2500 cycle PE sequencing chemistry (Illumina, San Diego, CA, USA). The Illumina Nextera XT kit was used for the library preparation following the standard protocol.

#### 4.2.6. Data Analysis and SNP Calling

The quality of raw sequencing data was checked using FastQC (v. 0.11.5) [40]. The raw reads were preprocessed using Trimmomatic (v. 0.36) [41] with the following settings: (a) very low qualities (Phred < 3) were removed from both the 5′ and 3′ ends; (b) low quality bases from the 3’ end were removed using a sliding window approach where we required average the Phred score of four consecutive bases to be at least 5; (c) reads shorter than 35 bp after the preprocessing were discarded; (d) unpaired reads were discarded as well. The preprocessed reads longer than 150 bp were mapped to a reference sequence using BWA MEM (v. 0.7.5a) [42]. Alignments were post-processed using SAMtools (v1.4). Alignments were discarded if (a) at least one read from a pair was unmapped, (b) reads were not mapped in a proper pair, (c) secondary alignments were detected, (d) non-primary alignments were detected or (e) multi-mapped reads were detected. Additional filtering was performed using NGSUtils/bamutils removeclipping and filter (v. 0.5.9) [43]. Alignments with more than 5% soft-clipped bases and alignments with more than 10% mismatches (calculated from the mapped read length) were discarded. An additional post-filtering requirement was a minimal read aligned length of 70 bp. Remaining alignments were indel realigned using the GATK (v. 3.5) [44], and duplicates were removed using Picard tools (v. 2.1.0) [45]. The resulting alignment quality and statistics were checked using the Qualimap (v. 2.1.3) [46] and custom R (v3.4.1) scripts.

Single nucleotide variants (both SNPs and Indels) were called using the GATK HaplotypeCaller (v. 3.5) [47], SAMtools (v. 1.4), and VarScan2 (v. 2.3.9) [48] according to published best practices recommend by developers of the tools. GATK (v. 3.5) base recalibration was applied during the variant calls. Raw variants were hard filtered using the recommended filters for each tool. After the variant calls, an overlap between the tools was created, and high confidence variants were extracted when there was an overlap of at least 2 tools. The number of bases at each variant site was obtained using bam-readcount (v. 0.8.0) [49], where only alignments with MAPQ at least 10 and Phred base quality of at least 15 were counted. The genotype information was extracted directly from the variant calls from individual tools using VCFtools (v. 0.1.13) [50]. Merged VCF files (VCFtools) were used for the visual inspection of variants. While the majority of sequences were analyzed using the above-mentioned pipeline, complex MHC DRB sequences were more difficult to resolve; therefore, SNP sites were checked and extracted manually for all individuals. Besides individual SNPs identified in the candidate genes, for groups of closely linked SNPs found in the same NGS reads, haplotypes could be determined.

#### 4.2.7. Population Characteristics of Polymorphic Markers: Stray Cats and the Study Group

For all SNPs identified in the genes studied, minor allele frequencies (MAFs) and heterozygosities were calculated for the cohort studied. These parameters were compared with those obtained for the group of stray cats (*n* = 38) representing a wide range of variation within the species.

### 4.3. Association Analyses

Chi-square and Fisher’s exact tests with Bonferroni corrections were used for assessing associations in all possible combinations of the phenotypes defined. Only SNPs and haplotypes with MAFs higher than 0.1 were used. The most significant associations (*p*_corrected_ < 0.01) out of those identified at *p*_uncorrected_ < 0.01 were further characterized and analyzed for gene–gene interactions using composed genotypes as previously described for other infections [51]. Only associations for which *p* values were at least 10 times lower than the lower of the two *p* values calculated for individual markers composing the genotype were considered. For SNPs associated with the phenotypes studied, Hardy–Weinberg equilibrium was determined using a web calculator [52].

## Figures and Tables

**Table 1:** Numbers of single nucleotide polymorphisms with Minor Allele Frequencies (MAFs) higher than 0.1 used for association analyses.

Gene	SNPs			Indels
	Total	Exonic	Non-Synonymous	Total
*IFNB1*	14	1	0	2
*IFNL1*	14	5	1	0
*IFNLR*	39	6	2	0
*NCR1*	29	4	1	6
*NCR2*	107	8	7	15
*NCR3*	7	0	0	2
*DRA*	3	3	0	0
*DRB*	47	47	37	4
*SNX5*	12	1	0	2
*SLX4IP*	3	0	0	1
*PAK5*	10	0	0	0
*PCSK2*	42	3	0	1
*LAMP5*	11	0	0	0

**Table 2:** Numbers of single nucleotide polymorphisms (SNPs) associated with Feline Enteric Coronavirus (FECV) shedding at *p*_uncorrected_ < 0.01.

Gene	SNPs Associated at *p* < 0.01		
	Shedding Pattern	Shedding Intensity	Shared
*IFNB1*	0	1	0
*IFNL1*	0	0	0
*IFNLR*	0	0	0
*NCR1*	0	7	0
*NCR2*	9	3	0
*NCR3*	1	0	0
*DRA*	1	1	0
*DRB*	2	1	1
*SNX5*	11	0	0
*SLX4IP*	3	3	3
*PAK5*	0	0	0
*PCSK2*	1	2	1
*LAMP5*	0	0	0

**Table 3:** Selected markers associated with different phenotypes at *p*_uncorrected_ < 0.01. Compared phenotype combinations are distinguished by numbers *.

Gene	Shedding Pattern (Polymorphisms/Haplotypes)						Shedding Intensity (Polymorphisms/Haplotypes)						Shared (Polymorphisms/Haplotypes)
Phenotypes Compared	1	2	3	4	5	6	7	8	9	10	11	12	
*IFNB1*											3/0		
*NCR1*					1/0		1/0	5/1	11/4	9/3	5/1		
*NCR2*	4/1	2/1	13/5	2/1	6/3	3/0	3/1	2/1	20/8	2/1	16/6	3/0	5/0
*NCR3*					1/0								
*DRA*		1/0	1/0					1/1	1/0		1/1		
*DRB*		1/0		2/0		1/0	1/0					1/0	1/0
*SNX5*	13/5		8/4		8/4								
*SLX4IP*			3/1		3/1		1/0	2/1	3/1	2/0	1/1		3/1
*PCSK2*					1/0				2/0	1/0	2/1		
	1—NS vs. PS					7—NS vs. HS							
	2—NS vs. IS					8—NS + VLS vs. HS							
	3—PS vs. IS					9—NS + VLS + LS vs. HS							
	4—NS vs. IS + US					10—NS + VLS + LS vs. MS + HS							
	5—PS vs. IS + US					11—NS + VLS + LS + MS vs. HS							
	6—NS vs. other phenotypes					12—NS vs. other phenotypes							

* The same SNP may be involved in several comparisons.

**Table 4:** SNPs with the most significant associations with FECV shedding.

GENE	*SLX4IP*		*NCR1*	
Position within the gene	174,768 **		3093	
p (uncorrected/corrected)	0.00065	0.0026	0.000067	0.0023
Comparison	PS vs. IS *		NS + VLS + LS vs. HS	
Position within the gene	175,354			
p (uncorrected/corrected)	0.00013	0.00054		
Comparison	PS vs. IS			
Position within the gene	175,575 **			
p (uncorrected/corrected)	0.00013	0.00054		
Comparison	PS vs. IS			
Position within the gene	174,768 **			
p (uncorrected/corrected)	0.0018	0.0072		
Comparison	PS vs. IS + US			
Position within the gene	175,354			
p (uncorrected/corrected)	0.0019	0.0074		
Comparison	PS vs. IS + US			
Position within the gene	175,575 **			
p (uncorrected/corrected)	0.00087	0.0035		
Comparison	PS vs. IS + US			
Position within the gene	175,354			
p (uncorrected/corrected)	0.0016	0.0065		
Comparison	NS + VLS + LS vs. HS			
Position within the gene	175,354			
p (uncorrected/corrected)	0.0014	0.0056		
Comparison	NS + VLS + LS + MS vs. HS			

* Shedding patterns. NS (Non-shedders): all four samples negative; PS (Persistent shedders): all four samples positive; IS (Intermittent shedders): at least one positive and one negative sampling followed by another positive sampling; US (Shedders with unclear status): shedding patterns could not be determined based on four samplings. Intensity of shedding. NS (Non-shedders): no virus detected; VLS (Very low-intensity shedders): 1–1000 virus particles; LS (Low-intensity shedders): 1000–4999 virus particles; MS (Medium-intensity shedders): 5000–99,999 virus particles; HS (High-intensity shedders): More than 100,000 virus particles. ** Loci out of HWE.

**Table 5:** Haplotypes with the most significant associations (*p* < 0.01) with FECV shedding.

GENE	*SNX5*	*NCR2*	*SLX4IP*	*NCR1*
Haplotype	*SNX5-D*	*NCR2-M*	*SLX4IP-A*	*NCR1-D*
SNP positions	15,736; 15,859	3030; 3227	1354; 1575	3093; 3248
p	0.0054	0.0059	0.0019	0.000067
Comparison	NS vs. PS *	NS + VLS + LS vs. HS	PS vs. IS + US	NS + VLS + LS vs. HS
Haplotype	*SNX5-A*	*NCR2-A*	*SLX4IP-A*	
SNP positions	16,219; 16,348	3031; 3227	1354; 1575	
p	0.0054	0.0058	0.00013	
Comparison	NS vs. PS	PS vs. IS	PS vs. IS	
Haplotype	*SNX5-B*	*NCR2-S*	*SLX4IP-A*	
SNP positions	16,988; 17,053; 17,057	3775; 3827	1354; 1575	
p	0.0054	0.0014	0.0039	
Comparison	NS vs. PS	PS vs. IS	NS + VLS + LS vs. HS	
Haplotype	*SNX5-C*		*SLX4IP-A*	
SNP positions	17,397; 17,429		1354; 1575	
p	0.0054		0.0014	
Comparison	NS vs. PS		NS + VLS + LS + MS vs. HS	
Haplotype	*SNX5-E*			
SNP positions	18,324; 18,563			
p	0.0054			
Comparison	NS vs. PS			
Haplotype	*SNX5-D*			
SNP positions	15,736; 15,859			
p	0.0052			
Comparison	PS vs. IS + US			

* Shedding patterns. NS (Non-shedders): all four samples negative; PS (Persistent shedders): all four samples positive; IS (Intermittent shedders): at least one positive and one negative sampling followed by another positive sampling; US (Shedders with unclear status): shedding patterns could not be determined based on four samplings. Intensity of shedding. NS (Non-shedders): no virus detected; VLS (Very low-intensity shedders): 1–1000 virus particles; LS (Low-intensity shedders): 1000–4999 virus particles; MS (Medium-intensity shedders): 5000–99,999 virus particles; HS (High-intensity shedders): More than 100,000 virus particles.

**Table 6:** Composed intragenic genotypes (IG) with the most significant associations with FECV shedding.

Genotype	Gene	SNPs	Phenotypes Compared	*p* (vs. Other Genotypes)
IG1	*NCR1*	2115; 3093	NS + VLS + LS vs. HS *	0.000067
IG2	*SLX4IP*	174,768; 175,354; 175,575	PS vs. IS	0.00013
IG3	*PCSK2*	271,166; 271,935	NS + VLS + LS vs. HS	0.0025
IG4	*DRB*	127; 155	NS vs. IS + US	0.0049

* Shedding patterns. NS (Non-shedders): all four samples negative; PS (Persistent shedders): all four samples positive; IS (Intermittent shedders): at least one positive and one negative sampling followed by another positive sampling; US (Shedders with unclear status): shedding patterns could not be determined based on four samplings. Intensity of shedding. NS (Non-shedders): no virus detected; VLS (Very low-intensity shedders): 1–1000 virus particles; LS (Low-intensity shedders): 1000–4999 virus particles; MS (Medium-intensity shedders): 5000–99,999 virus particles; HS (High-intensity shedders): More than 100,000 virus particles.

**Table 7:** Selected composed intergenic genotypes (CG) with the most significant associations with FECV shedding.

Genotype	Gene 1	SNP 1	Gene 2	SNP 2	Phenotypes Compared	*p*
CG1	*PCSK2*	271,935	*SLX4IP*	175,575	PS vs. IS + US *	0.000047
CG2	*PCSK2*	271,935	*SNX5*	18,563	PS vs. IS + US	0.00025
CG5	*SNX5*	15,736	*SLX4IP*	175,575	PS vs. IS	0.000042
CG6	*NCR1*	3093	*NCR2*	1030	NS vs. HS	0.0082
CG7	*NCR1*	3093	*NCR2*	871	NS + VLS vs. HS	0.00071
CG8	*NCR1*	3093	*NCR2*	3030	NS + VLS + LS vs. HS	0.00046

* Shedding patterns. NS (Non-shedders): all four samples negative; PS (Persistent shedders): all four samples positive; IS (Intermittent shedders): at least one positive and one negative sampling followed by another positive sampling; US (Shedders with unclear status): shedding patterns could not be determined based on four samplings. Intensity of shedding. NS (Non-shedders): no virus detected; VLS (Very low-intensity shedders): 1–1000 virus particles; LS (Low-intensity shedders): 1000–4999 virus particles; MS (Medium-intensity shedders): 5000–99,999 virus particles; HS (High-intensity shedders): More than 100,000 virus particles.

## Supplementary Materials

The following are available online at https://www.mdpi.com/2076-0817/9/11/958/s1. Table S1: Population characteristics. Table S2: Associations in British Shorthair and Maine Coon. Table S3: Haplotypes. Table S4: SNPs associated before Bonferroni correction (+HWE in these loci). Table S5: SNPs associated after Bonferroni correction (+HWE in these loci). Table S6: Composed genotypes. Table S7: Alleles/genotypes associated with susceptibility/resistance to FECV shedding. Table S8: Shedding intensity. Table S9: List of breeds. Table S10: Candidate genes. Table S11: Designed primers. Table S12: Amplified genes and regions. Table S13: Polymerases used for PCR amplifications. Raw data (Excel files): available online in Mendeley repository https://data.mendeley.com/datasets/cxx68cw77c/draft?a=ac846404-9385-424e-890f-62484981eb64.
